# Homozygous *HESX1* and *COL1A1* Gene Variants in a Boy with Growth Hormone Deficiency and Early Onset Osteoporosis

**DOI:** 10.3390/ijms22020750

**Published:** 2021-01-13

**Authors:** Viola Alesi, Maria Lisa Dentici, Silvia Genovese, Sara Loddo, Emanuele Bellacchio, Valeria Orlando, Silvia Di Tommaso, Giorgia Catino, Chiara Calacci, Giusy Calvieri, Daniele Pompili, Graziamaria Ubertini, Bruno Dallapiccola, Rossella Capolino, Antonio Novelli

**Affiliations:** 1 Laboratory of Medical Genetics, Translational Cytogenomics Research Unit, Bambino Gesù Children Hospital, IRCCS, 00146 Rome, Italy; silvia.genovese@opbg.net (S.G.); sara.loddo@opbg.net (S.L.); valeria.orlando@opbg.net (V.O.); silvia.ditommaso@opbg.net (S.D.T.); giorgia_14@hotmail.it (G.C.); chiara.calacci@opbg.net (C.C.); giusy.calvieri@opbg.net (G.C.); daniele.pompili@opbg.net (D.P.); antonio.novelli@opbg.net (A.N.); 2 Medical Genetics Unit, Bambino Gesù Children Hospital, IRCCS, 00146 Rome, Italy; marialisa.dentici@opbg.net (M.L.D.); bruno.dallapiccola@opbg.net (B.D.); rossella.capolino@opbg.net (R.C.); 3 Department of Research Laboratories, Bambino Gesù Children Hospital, IRCCS, 00146 Rome, Italy; Emanuele.Bellacchio@OPBG.net; 4 Endocrine Unit, Bambino Gesù Children Hospital, IRCCS, 00146 Rome, Italy; graziamaria.ubertini@opbg.net

**Keywords:** *HESX1*, Q6H, *COL1A1*, E1361K, osteoporosis, combined pituitary hormone deficiency, CPHD

## Abstract

We report on a patient born to consanguineous parents, presenting with Growth Hormone Deficiency (GHD) and osteoporosis. SNP-array analysis and exome sequencing disclosed long contiguous stretches of homozygosity and two distinct homozygous variants in *HESX1* (Q6H) and *COL1A1* (E1361K) genes. The *HESX1* variant was described as causative in a few subjects with an incompletely penetrant dominant form of combined pituitary hormone deficiency (CPHD). The *COL1A1* variant is rare, and so far it has never been found in a homozygous form. Segregation analysis showed that both variants were inherited from heterozygous unaffected parents. Present results further elucidate the inheritance pattern of *HESX1* variants and recommend assessing the clinical impact of variants located in C-terminal propeptide of *COL1A1* gene for their potential association with rare recessive and early onset forms of osteoporosis.

## 1. Introduction

Chromosomal Microarrays Analysis (CMA) and whole exome sequencing (WES) are getting more and more important in the diagnostic setting and are particularly useful in addressing genetically heterogeneous disorders. They are also important for patients presenting with non-specific clinical features, or who display complex phenotypes resulting from the co-occurrence of two or more rare diseases.

In the case of parental inbreeding, the combined use of these techniques provides a homozygosity-by-descent mapping to search for candidate genes and allows the detection of causative variants [1,2].

We report the case of a boy, born to consanguineous parents, presenting with a severe short stature with a growth hormone deficiency, psychomotor delay, corpus callosum hypoplasia, low-grade glioma, spastic paraparesis, and osteoporosis.

Mutations in the transcription factor HESX1 can cause isolated growth hormone deficiency (IGHD) or combined pituitary hormone deficiency (CPHD) with or without septo-optic dysplasia (SOD). CPHD is a severe disorder characterized by combined deficiency of growth hormone (GH) and at least one additional pituitary hormone. It is associated with variable clinical manifestations depending on the patient’s age and hormone residual levels [3]. Developmental delay, growth retardation with an increased weight-to-height ratio, hypoglycemia, jaundice, genitourinary abnormalities, pubertal development failure, hypothyroidism, and midline defects are often observed in affected patients [4]. So far, eight genes have been associated with CPHD (OMIM https://www.omim.org/), including *LHX4* (1q25.2), *GLI2* (2q14.2), *HESX1* (3p14.3), *POU1F1* (3p11.2), *PROP1* (5q35.3), *LHX3* (9q34.3), *OTX2* (14q22.3), and *SOX3* (Xq27.1). Mutations in early transcription factors, such as *HESX1* and *GLI2*, may result in extra-pituitary manifestations, including septo-optic dysplasia (SOD) and holoprosencephaly (HPE) [5]. No association with a distinct skeletal phenotype has so far been reported.

Osteoporosis is a chronic skeletal disease characterized by low bone mass and bone mineral density (BMD). It is associated with an imbalanced bone formation/resorption or with an abnormal matrix deposition, leading to bone microarchitecture disruption, compromised bone strength, and a high propensity for low-energy fractures in long bones and vertebrae [6].

In the present patient, a combined SNP-array and exome-sequencing approach led to the identification of two homozygous variants in *HESX1* and *COL1A1* genes, thus unravelling the molecular defects underlying his complex phenotype.

### Clinical Description

The proband, a 13-year-old Egyptian boy, is the third living child of healthy double first cousins parents (the maternal grandmother and paternal grandfather are siblings, and so are the maternal grandfather with the paternal grandmother). The parents had a total of 7 pregnancies. The first child was a female affected by situs viscerum inversus and unspecified congenital heart disease, who died at age of 8 months. The second pregnancy ended with the spontaneous demise of a male fetus with hydrocephalus and limbs’ malformation at the 7th month of gestational age. The third and fourth newborns were healthy females, now 21 and 18 years old. The fifth and seventh pregnancies ended with first trimester spontaneous abortions.

**Figure 1:**
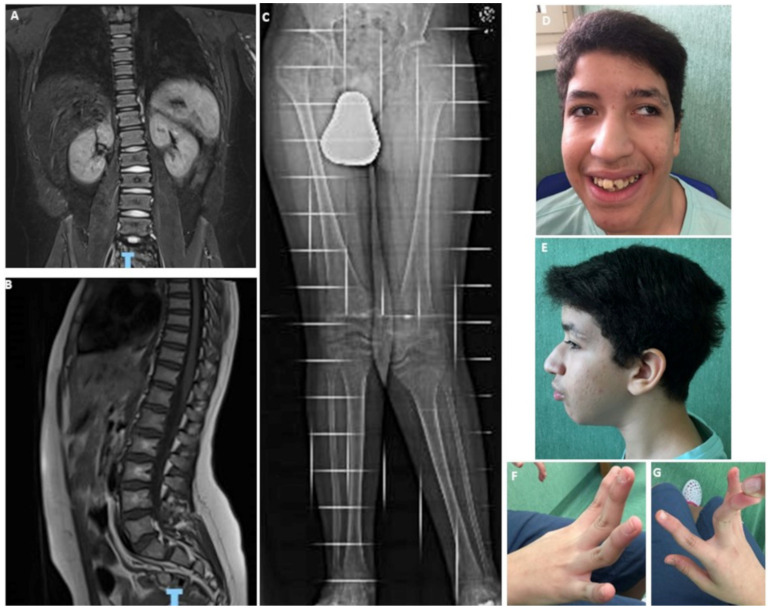
(**A**,**B**) MRI of lumbosacral spine at 13 years. Note the altered morphology of the vertebral bodies, which have a reduced height secondary to non-recent vertebral compression fractures, more marked in L5. The vertebral canal has a regular diameter. (**C**) X-ray of lower limbs showing right hip subluxation and a severely valgus left knee. (**D**–**G**) Physical examination of the patient at 13 years old, showing divergent strabismus, open mouth appearance, large and malpositioned teeth, hypermobility of distal interphalangeal joints.

The pregnancy of the present patient was uneventful, and birth at term with a spontaneous vaginal delivery. Birth weight was 3800 g (75th centile). Respiratory distress was reported in the perinatal period requesting 1-month hospitalization in neonatal intensive care unit. Orchidopexy for right cryptorchidism was performed at 2 years. Bilateral cataracts were surgically removed at 6 years. Developmental milestones were grossly delayed. Unsupported walking, with a still present waddling gait, was achieved at 42 months. His first words were pronounced after age 4. He has not yet acquired bladder control. At 13 years old, a Vineland Adaptive Behavior Scales-II revealed an IQ of 60. Cerebral MRI at 9 and 13 years disclosed hypoplastic and dysmorphic corpus callosum, bilateral parietal-frontal abnormalities of the white matter with punctiform gliosis, an 8 mm area of hyperintensity on the right cerebral peduncle with high levels of diffusion in the apparent diffusion coefficient (ADC) maps, suggesting a low grade glioma (LGG), which is regularly monitored by neuroradiologists and oncologists. MRI of pituitary gland was normal. Two-dimensional color-doppler echocardiography was unremarkable. At 13 years, neurogenic club foot was surgically corrected. An X-ray at 11 years and 4 months revealed a mild left convex scoliosis in the dorsal and lumbar spine (T10-L4) of 10 degrees. Generalized platyspondyly of the dorsal vertebral bodies with wedge deformity of the anterior vertebral bodies of T8-T11 and biconcave lens deformity of the lumbar vertebrae were also found, and regarded as multiple vertebral collapses, secondary to osteoporotic fractures (Figure 1). MRI of lumbosacral spine at 13 years disclosed an altered shape of all vertebral bodies, which showed a reduced height suggesting a non-recent collapse (vertebral compression fractures), more pronounced at L5. The vertebral canal had a regular diameter. Lower limbs X-ray displayed subluxation of the right hip, with a pelvic difference of 15 mm at left, and a severely valgus left knee.

At 13.2 years, weight was 36.5 kg (−1.53 SD), height 130.5 cm (<3rd centile; −3.65 SD), and OFC 51.5 cm (<3rd centile). The height velocity was 2.8 cm per year (−3.62 SD).

Clinical examination disclosed divergent strabismus, nystagmus, open mouth, large and malpositioned teeth, small thorax, a supernumerary nipple, single transverse crease on the left palm, lower limbs heterometry, and hypermobility of distal interphalangeal joints. No signs of blue sclerae or of dentinogenesis imperfecta were noticed. Neurological examination revealed spasticity hypertonia at the 4 limbs (lower limbs > than upper limbs), bilateral Babinski reflex, surgically treated neurogenic club foot, and stereotyped movements with hand flapping and body rocking. Puberty was markedly delayed, and at 13 years old he disclosed Tanner stages P2, G1, bilateral testicular volume of 3–4 mL, and microphallus. Testis were undescended and the left one can be felt in the inguinal canal.

The hypothalamic-pituitary-thyroid axis was normal with TSH of 2.11 mcUI/mL (normal value: 0.51–4.94) and FT4 of 1.13 ng/dl (n.v.: 0.89–1.76).

ACTH test (with 250 mcg of Cortrosyn) revealed normal pituitary function of pituitary-adrenal axis while Arginine and Clonidine test disclosed GH deficiency. The GH peak after Arginine was 1.9 ng/mL and after Clonidine was 4.34 ng/mL, respectively. IGF-1 levels were below the normal range for age (149 ng/mL; n.v. 177.0–507.0). The patient has never taken GH therapy for the presence of glioma at MRI.

Testosterone levels were below the normal range for his age (33.2 ng/dL; n.v. 33.3–742.5), with normal response of gonadotropins to LHRH test suggesting a delay of puberty.

Vitamin D3 25 OH levels were subnormal (17.5 < ng/mL; n.v. 20.0–120.0) with normal value of calcium (10.1 mg/dL; n.v. 8.5–10.50), phosphorus (4.9 mg/dL; n.v. 2.7–4.5), PTH (52 pg/mL; n.v. 18.5–88), and alkaline phosphate (707 UI/L; n.v. 177–938). Bone density scan (DEXA scan) was performed at 12 and 13 years old, revealing a progressive reduced bone mineral density (BMD) with a *Z* score of −2.4. and −2.6, respectively. He is taking cholecalciferol at the dosage of 5000 UI orally a week. Karyotype and targeted NGS of spastic paraplegia genes were unremarkable.

## 2. Results

SNP-array analysis in the patient showed no copy number variants at a resolution of 100 Kb. Several long contiguous stretches of homozygosity (LCSH) where detected along the genome, accounting for 16.88% of the autosomal sequence. This high rate likely reflects the double parents’ consanguinity and their origin from the same ethnic isolate and suggested that the patient was affected by a recessive disorder.

Whole exome sequencing was performed with a main focus on the LCSH regions, looking for homozygous causative variants. A homozygous missense variant was detected in *HESX1* gene, consisting in a glutamine versus histidine substitution in exon 1: NM_003865.2 c.[18G>C];[18G>C] NP_003856.1 (p.[Gln6His];[Gln6His]). This variant, in heterozygous status, has been causally associated with an incompletely penetrant dominant form of CPHD [7], while no homozygous individual has been reported so far.

**Figure 2:**
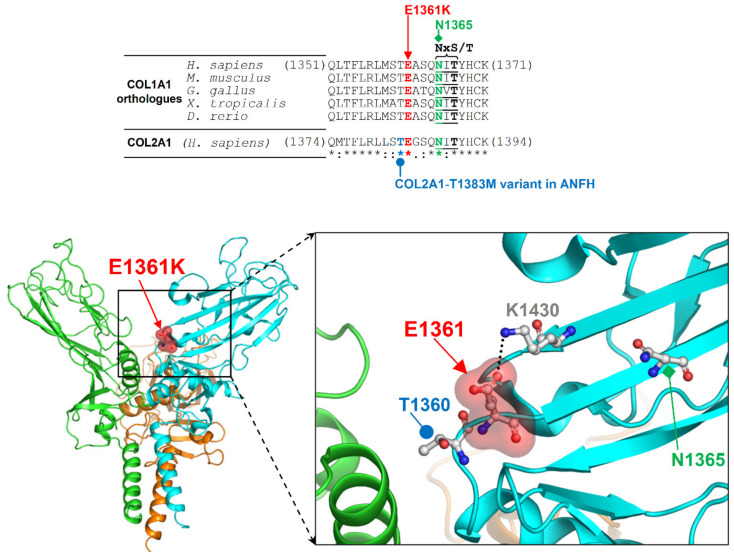
Sequence alignment and structure around the site of *COL1A1* E1361K variant. Top: Multiple sequence alignment around E1361 among orthologues of COL1A1 and its human paralogue COL2A1; residue conservation is indicated by symbols below the alignment (“*” fully conserved, “:” strongly conserved, and “.” weakly conserved); consensus residues NxS/T (where “x” can be any residue except proline) for the *N*-glycosylation of N1365 are underscored. Bottom: Crystal structure of homotrimer of human fibrillar procollagen type I C-propeptide (PDB entry 5K31; the three protein monomers are in different ribbon colors); the closed-up view shows E1361 together with its salt-bridge forming partner K1430, the potentially glycosylable N1365, and T1360 that corresponds to the threonine affected by the T1383M variant in COL2A1 previously reported in a patient with avascular necrosis of femoral head; amino acid numbering of COL1A1 refers to the NCBI protein entry NP_000079.2.

A second homozygous variant was detected in *COL1A1* gene, consisting in a glutamate versus lysine substitution in exon 50: NM_000088.3 c.[4081G>A];[4081G>A], NP_000079.2 (p.[Glu1361Lys];[Glu1361Lys]). This variant (Osteogenesis Imperfecta variant Database AN_000141) is regarded as a Variant of Unknown Significance (ClinVar rs141011435) and reported in Exac and gnomAD browsers with a frequency lower than 1/20,000. No homozygous subjects have been reported so far. The E1361K variant is localized in a conserved loop of the *C*-terminal propeptide, near the interface of interaction with another COL1A1 molecule shown by the crystal structure of homotrimerized protein [8]. The Glu1361 residue affected by this mutation contributes to the local structure by forming a salt-bridge with Lys1430 and is also close to a region matching the NxS/T consensus motif for the *N*-linked glycosylation of Asn1365 (Figure 2). Thus, we expect that the E1361K variant affects the homo- and hetero-trimerization of collagen and the potential glycosylation of Asn1365. Furthermore, Glu1361 is adjacent to Thr1360, which corresponds to Thr1383 in the paralogue COL2A1. The COL2A1 T1383M variant was found in a patient with avascular necrosis of the femoral head [9], supporting the pathogenicity of the novel E1361K variant (Figure 2).

Segregation analysis disclosed that both gene variants were inherited from the heterozygous healthy parents and were also present in the sisters. No additional known high impact variants were detected.

## 3. Discussion

*HESX1* encodes a conserved homeobox protein acting as transcriptional repressor in the developing forebrain and pituitary gland. Its expression, initially involving both the anterior midline visceral endoderm and the neural ectoderm, is then restricted to the Rathke pouch. *HESX1* is required also for the development of forebrain, eyes, and other anterior structures, such as olfactory placodes (UniProtKB-Q9UBX0, https://www.uniprot.org/). The clinical consequences of *HESX1* mutations depend on the variant type, their effects on the final protein and, supposedly, on modifier genes or environmental factors [10,11,12,13]. Carrier patients manifest either isolated GH deficiency, CPHD, or septo-optic dysplasia (SOD). Causative variants have been described both in heterozygous and in homozygous individuals, suggesting either dominant or recessive inheritance of the associated disorder, the heterozygous carriers presenting with a milder phenotype [14].

Here, we report on a boy born to consanguineous parents, presenting with a complex phenotype characterized by psychomotor delay, corpus callosum hypoplasia, low-grade glioma in the right cerebral peduncle, spastic paraparesis, osteoporosis, scoliosis, pubertal retardation, and GHD. A homozygous causative missense variant in *HESX1* gene was detected, resulting in a Q6H substitution. This heterozygous variant has been reported in 3 patients presenting with anterior pituitary hormone deficiency (Table 1) [7,15,16]. In two patients, the variant was inherited from an unaffected parent, while parents were not tested in one. Based on results from knockout mice an incompletely penetrant dominant model was proposed [14], although a compound heterozygous condition was not ruled out [17]. Homozygous variants in *HESX1* have been reported in SOD [11]. This disorder was excluded in our patient, for the absence of distinct brain malformations, including optic nerve hypoplasia, pituitary gland hypoplasia, and agenesis of midline brain structures, although he has corpus callosum hypoplasia.

In order to further investigate the impact of the variant, analysis of family segregation was carried out disclosing that both healthy parents and the sisters were heterozygous for the c.18G > C *HESX1* variant, suggesting an autosomal recessive model.

The concurrent homozygous missense variant in *COL1A1* argued for its pathogenic role of the patient’s skeletal features. Causative heterozygous variants in *COL1A1* are associated with Osteogenesis Imperfecta, a rare connective disorder, characterized by a highly variable phenotype, ranging from moderate to lethal forms. Clinical features include increased susceptibility to bone fractures, reduced bone density, short stature, scoliosis and skull deformity, hearing and sight impairment, dentinogenesis imperfecta and blue sclerae. The rare *COL1A1* homozygous variant detected in our patient results in an E1361K substitution at the *C*-terminal propeptide involved in α-chain selection and folding. This terminal propeptide is absent in mature collagen fibrils, being processed before their assembly, but it is critical for α-chain alignment and recognition during procollagen synthesis, for obtaining a proper 2:1 α-chain composition [18]. Mutations in the *C*-terminal procollagen cause delay in α-chain incorporation, slow folding and overmodification, resulting in procollagen accumulation, reduced production of mature collagen, abnormal osteoblast differentiation, and defective matrix function. Causative heterozygous variants in this domain occur in 6.5% of OI patients [18,19].

To date, no homozygous variant has ever been reported in the *COL1A1* gene, while biallelic *COL1A2* variants have been described in individuals with autosomal recessive OI [20,21,22,23,24,25,26,27]. In these families, the heterozygous carriers were either healthy or mildly affected, while the four adult subjects heterozygous for the new *COL1A1* variant in our family were all healthy. The present family suggests that biallelic *COL1A1* mutations can lead to a skeletal involvement mainly characterized by a severe and early onset osteoporosis, without the extra-skeletal manifestation of OI.

In conclusion, this study further elucidates the inheritance of *HESX1* variants and recommends assessing the clinical impact of variants located in the *C*-terminal propetide of *COL1A1* gene for their potential association with rare recessive forms of osteoporosis.

## 4. Materials and Methods

Clinical data were obtained in accordance with the ethical standards of the Ospedale Pediatrico Bambino Gesù (Rome, Italy) review board (RRC-2018-2365812.). Informed consent was signed by patient’s parents. The DNA of proband and family members was isolated from peripheral blood by a QIAsymphony automatic extractor (QIAGEN, www.qiagen.com).

### 4.1. SNP-Array Analysis (Single Nucleotide Polymorphism–Array Analysis)

SNP-array was performed on patient’s DNA in accordance with the manufacturer’s instructions, using Infinium CytoSNP−850 K BeadChip (Illumina, San Diego, CA). Array scanning data were generated by the iScan system (Illumina) and the results were analyzed by Bluefuse Multi software.

### 4.2. Target Exome Sequencing

Whole exome capture was performed on the patient’s DNA by using the high-throughput NimbleGen SeqCap Exome Enrichment kit (Roche https://www.roche.com/) according to the manufacture’s protocol and sequenced on an Illumina NextSeq 550 platform. Sequencing data alignment to the hg19 human reference genome and variant calling were performed by BWA Genome Alignment Software and GATK Variant Caller (Illumina). Annotating and filtering were performed by Variant Studio software (Illumina, http://variantstudio.software.illumina.com/) and Geneyx Analysis software (formerly TGex) (https://pubmed.ncbi.nlm.nih.gov/31888639/). The variants, identified as pathogenic, were confirmed by Sanger sequencing using a standard protocol (BigDye Terminator v3.1 Cycle Sequencing Kit, Applied Biosystems by Life Technologies). Sequencing was extended to parents and two patient’s sisters for assessing the segregation of variants.

## Figures and Tables

**Table 1:** Clinical details of patients harboring 18G > C variant in *HESX1.*

Authors	Status Variant (18G > C, Q6H)	Family Unaffected Heterozygous Carriers	Pituitary Gland	GH	TSH	ACTH	LH/FSH	Optic Nerve	Corpus Callosum Septum Pellucidum	Other Clinical Features
Thomas 2001	Heterozygous	Father	Anterior pituitary hypoplasia and ectopic posterios pituitary	Deficient	Deficient by age of 7 years	Normal	Deficient	Normal	Normal	Small penis
Corneli 2007 (also described by Vivenza 2011 and De Rienzo 2015)	Heterozygous	Mother	Anterior pituitary hypoplasia and ectopic posterior pituitary	Deficient	Deficient	Deficient	Prepubertal at diagnosis	Normal	Normal	Narrow biparietal diameter, mandibular hypoplasia, bilateral cryptorchidism, micropenis
Newbern 2013	Heterozygous	Unknown	Not reported	Not reported	Not reported	Not reported	Not reported	Not reported	Not reported	Idiopathic hypogonadotropic hypogonadism, anosmia
Our patient	Homozygous	Father, mother, two sisters	Normal	Deficient	Normal	Normal	Normal	Normal	Corpus callosum hypoplasia	Low-grade glioma in the right cerebral peduncle, spastic paraparesis, osteoporosis, scoliosis, pubertal retardation

## Data Availability

The data presented in this study are available on request from the corresponding author. The data are not publicly available due to privacy and ethical restrictions.
